# A colorimetric RT-LAMP assay and LAMP-sequencing for detecting SARS-CoV-2 RNA in clinical samples

**DOI:** 10.1126/scitranslmed.abc7075

**Published:** 2020-08-12

**Authors:** Viet Loan Dao Thi, Konrad Herbst, Kathleen Boerner, Matthias Meurer, Lukas PM Kremer, Daniel Kirrmaier, Andrew Freistaedter, Dimitrios Papagiannidis, Carla Galmozzi, Megan L. Stanifer, Steeve Boulant, Steffen Klein, Petr Chlanda, Dina Khalid, Isabel Barreto Miranda, Paul Schnitzler, Hans-Georg Kräusslich, Michael Knop, Simon Anders

**Affiliations:** 1 Schaller Research Groups, Department of Infectious Diseases, Virology, Heidelberg University, Heidelberg, Germany.; 2 Department of Infectious Diseases, Virology, Heidelberg University, Heidelberg, Germany.; 3 Center for Molecular Biology of Heidelberg University (ZMBH), Heidelberg, Germany.; 4 German Center for Infection Research (DZIF), Heidelberg, Germany.; 5 German Cancer Research Center (DKFZ), Heidelberg, Germany.; 6 DKFZ-ZMBH Alliance, Heidelberg, Germany.

## Abstract

The coronavirus disease 2019 (COVID-19) pandemic caused by the SARS-CoV-2 (severe acute respiratory syndrome coronavirus 2) coronavirus is a major public health challenge. Rapid tests for detecting existing SARS-CoV-2 infections and assessing virus spread are critical. Approaches to detect viral RNA based on reverse transcription loop-mediated isothermal amplification (RT-LAMP) have potential as simple, scalable, and broadly applicable testing methods. Compared to RT quantitative polymerase chain reaction (RT-qPCR)–based methods, RT-LAMP assays require incubation at a constant temperature, thus eliminating the need for sophisticated instrumentation. Here, we tested a two-color RT-LAMP assay protocol for detecting SARS-CoV-2 viral RNA using a primer set specific for the *N* gene. We tested our RT-LAMP assay on surplus RNA samples isolated from 768 pharyngeal swab specimens collected from individuals being tested for COVID-19. We determined the sensitivity and specificity of the RT-LAMP assay for detecting SARS-CoV-2 viral RNA. Compared to an RT-qPCR assay using a sensitive primer set, we found that the RT-LAMP assay reliably detected SARS-CoV-2 RNA with an RT-qPCR cycle threshold (CT) number of up to 30, with a sensitivity of 97.5% and a specificity of 99.7%. We also developed a swab–to–RT-LAMP assay that did not require a prior RNA isolation step, which retained excellent specificity (99.5%) but showed lower sensitivity (86% for CT < 30) than the RT-LAMP assay. In addition, we developed a multiplexed sequencing protocol (LAMP-sequencing) as a diagnostic validation procedure to detect and record the outcome of RT-LAMP reactions.

## A colorful test for SARS-CoV-2 RNA detection

We need simple methods to rapidly test large numbers of people for infection with the SARS-CoV-2 coronavirus. Quantitative PCR (qPCR) after reverse transcription (RT), the standard method, is very sensitive but requires expensive instrumentation. Loop-mediated isothermal amplification (LAMP) is an alternative to qPCR that is faster and requires fewer resources. Dao Thi *et al.* tested the RT-LAMP assay on several hundred clinical RNA samples isolated from pharyngeal swabs collected from individuals being tested for COVID-19. They confirmed that the RT-LAMP assay was a simpler albeit less sensitive option compared to RT-qPCR for large-scale testing for SARS-CoV-2 RNA. These investigators also developed a simplified version of this method (direct swab–to–RT-LAMP assay) that did not require a prior RNA isolation step as well as a method for highly multiplexed sequencing of RT-LAMP reactions (LAMP-sequencing).

## Teaser

A colorimetric isothermal RNA amplification method was shown to detect SARS-CoV-2 RNA in clinical samples with excellent sensitivity and specificity.

## INTRODUCTION

The coronavirus disease 2019 (COVID-19) pandemic, caused by the SARS-CoV-2 (severe acute respiratory syndrome coronavirus 2) coronavirus (*1*), is a major global health threat. A still unknown proportion of people, especially the elderly and those with preexisting conditions, are at high risk of a severe course of COVID-19 (*2*), leading to a high burden on health care systems worldwide. Further, because of limited testing capacity, only people with symptoms are usually tested for SARS-CoV-2 infection, although studies have confirmed that many individuals infected with SARS-CoV-2 are asymptomatic carriers of the virus (*3*, *4*). This suggests that infection control strategies focusing on symptomatic patients are not sufficient to prevent virus spread.

Therefore, large-scale diagnostic methods are needed to determine the spread of the virus in populations quickly, comprehensively, and sensitively. This would allow for the rapid isolation of infected persons during an existing wave of infection. In addition, continuous and repeated testing of large groups within a population may be required as a long-term strategy to contain new outbreaks while keeping societies and economies functional until effective vaccines become available.

An active SARS-CoV-2 infection can be diagnosed by detecting either the viral genome or viral antigens in appropriate human samples. Assays for detecting SARS-CoV-2 antigens are limited by the sensitivity, specificity, and production speed of diagnostic antibodies, whereas detecting viral RNA only requires specific oligonucleotides. Therefore, an assay that detects SARS-CoV-2 RNA facilitates testing of large cohorts.

The SARS-CoV-2 diagnostic pipeline that has proven to be successful and that is currently used in many test centers consists of three steps: collecting nasopharyngeal or oropharyngeal swab specimens, isolation of total RNA, and specific detection of the viral genome by RT-qPCR. The latter comprises a reverse transcriptase (RT) step, which translates the viral RNA into DNA, followed by a semiquantitative DNA polymerase chain reaction using oligonucleotides specific for the viral cDNA (qPCR). As a result, a short piece of the viral genome is strongly amplified and then is detected by a sequence-specific oligonucleotide probe labeled with a fluorescent dye.

This procedure includes several steps that require sample handling; therefore, the detection process in a clinical diagnostic laboratory takes about 3 to 24 hours or more, depending on the number of samples and process optimization of the test center. In addition, in the context of the COVID-19 pandemic, many of the reagents required are only slowly being replenished due to insufficient production capacity or lack of international transport. Therefore, increasing daily test capacities for RT-qPCR–based diagnostics for SARS-CoV-2 RNA detection is currently limited. To accelerate and optimize such diagnostics, new scalable methods for RNA isolation and the detection of viral genomes are needed.

An alternative to RT-qPCR is reverse transcription loop-mediated isothermal amplification (RT-LAMP) (*5*–*7*). RT-LAMP reactions include a reverse transcriptase and a DNA polymerase with strong strand displacement activity and tolerance for elevated temperatures and up to six DNA oligonucleotides of a certain architecture. Samples with potential template molecules are added to the reaction and incubated for 20 to 60 min at a constant temperature (e.g., 65°C). The oligonucleotides act as primers for the reverse transcriptase, and additional oligonucleotides for the DNA polymerase are designed so the DNA products loop back at their ends. These, in turn, serve as self-priming templates for the DNA polymerase. In the presence of a few RNA template molecules, a chain reaction is set in motion, which then runs until the added reagents (in particular, the deoxynucleotide triphosphates) are used up.

To detect DNA production in RT-LAMP assays, various approaches have been described. One possibility is to use a pH indicator (e.g., phenol red) and run the reaction in a weakly buffered environment. As the chain reaction proceeds, the pH is lowered, which results in a visible color change from red to yellow making it an appealing assay for point-of-care diagnosis (*8*). Previously, RT-LAMP assays have been proposed for diagnostic detection of other RNA viruses, such as influenza virus (*9*). Also, several studies have demonstrated the use of isothermal DNA amplification to detect small amounts of SARS-CoV-2 RNA. The majority of these studies used in vitro transcribed (IVT) short fragments of the viral genomic RNA (*10*–*12*) and showed a detection limit of somewhere between 10 and 100 RNA molecules per reaction. For the detection of SARS-CoV-2 RNA, a few commercial rapid tests have been developed [reviewed in (*13*)] using isothermal DNA amplification reactions involving proprietary enzyme formulations that are not commercially available in a ready-to-go format. Further, their exact sensitivity is still subject to discussion owing to a lack of studies using sufficiently large numbers of test samples.

The performance of an RT-LAMP assay does not require expensive special equipment such as a thermal cycler with real-time fluorescence measurement, because positive samples are determined by a color change from red to yellow within 30 min after the start of the incubation at 65°C. For detection, simple mobile phone cameras, copy machines, office scanners, or plate scanners with spectrophotometric quantification can be used. During the early phase of the COVID-19 pandemic (early March 2020) in Germany, we tested the sensitivity and specificity of a colorimetric RT-LAMP assay for detecting SARS-CoV-2 RNA in clinical RNA samples isolated from pharyngeal swab specimens collected from individuals being tested for COVID-19 (and provided by the Heidelberg University Hospital’s diagnostic laboratory after removal of an aliquot for SARS-CoV-2 RNA testing by RT-qPCR) (fig. S1). We also developed a swab–to–RT-LAMP assay that used naso/oropharyngeal swab specimens directly without the need for an RNA isolation step. We tested >700 clinical RNA samples with a wide range of viral loads, allowing us to determine accurately the sensitivity range of the colorimetric RT-LAMP assay. We also developed a multiplexed LAMP-sequencing protocol using barcoded Tn5 transposase tagmentation that enabled rapid identification of positive results in thousands of RT-LAMP reactions within the same next-generation sequencing run.

## RESULTS

### Establishing colorimetric RT-LAMP assay sensitivity using an artificial SARS-CoV-2 RNA template

**Fig. 1:**
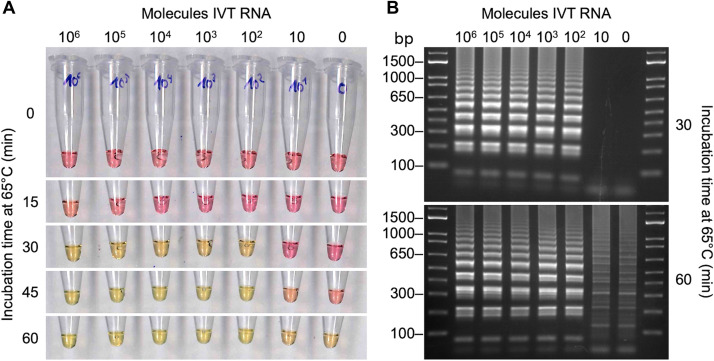
Sensitivity of the RT-LAMP assay determined using IVT RNA. (**A**) Defined numbers of in vitro transcribed (IVT) RNA molecules of the SARS-CoV-2 *N* gene were added to the RT-LAMP reaction and incubated at 65°C. At indicated times, samples were removed from the heating block and cooled on ice to stop the reaction. Photographs were taken using the color scanner function of an office copy machine and show the red to yellow color change in positive samples. (**B**) The RT-LAMP reaction product (2.5 μl) was analyzed on a 2% agarose gel. The typical band pattern of a successful RT-LAMP reaction was visible in the samples with 100 or more SARS-CoV-2 RNA molecules, i.e., in those samples that showed a color change from red to yellow after 30 min.

To detect SARS-CoV-2 RNA with RT-LAMP, we used the WarmStart Colorimetric RT-LAMP 2X Master Mix (DNA and RNA) from New England Biolabs. This mix contains two enzymes, an engineered reverse transcriptase (RTx) and a strand-displacing polymerase (Bst 2.0). In addition, the reaction mixture contains oligonucleotide-based aptamers that function as reversible temperature-dependent inhibitors, ensuring that the reaction only runs at an elevated temperature (WarmStart) to avoid nonspecific priming reactions. Several primer sets were recently proposed for RT-LAMP–based detection of SARS-CoV-2 RNA by Zhang *et al. (**11*) and by Yu *et al. (**10*), and these primer sets were subsequently validated with in vitro–translated RNA. We prepared and tested two primer sets for different RNA sections of the SARS-CoV-2 genome, the N-A set targeting the *N* gene and the 1a-A set targeting open reading frame (ORF) 1a (table S1) (*11*). Figure 1A shows that the oligonucleotide set for the *N* gene was capable of detecting 100 IVT RNA molecules in a test reaction with 1 μl of RNA solution, as evidenced by the red-to-yellow color change. The reaction was conducted for up to 1 hour at 65°C. For time points > 30 to 35 min, the negative control frequently became yellowish (Fig. 1A). This was caused by spurious amplification products, which is a well-known problem with RT-LAMP (*14**).* Analysis by gel electrophoresis revealed clearly distinct banding patterns for the correct RT-LAMP reaction products (lanes with ≥100 molecules IVT RNA input) and the spurious reaction products (Fig. 1B).

### Testing clinical RNA samples with the colorimetric RT-LAMP assay

**Fig. 2:**
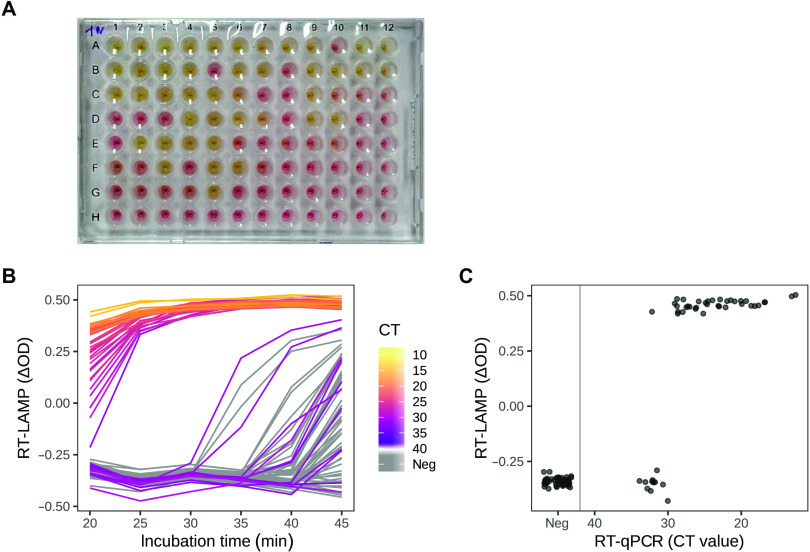
Sensitivity and specificity of the RT-LAMP assay compared to RT-qPCR using clinical samples. RNA samples isolated from 95 pharyngeal swab specimens were analyzed by the RT-LAMP assay using a 96-well plate. The RT-LAMP reaction was incubated at 65°C, and the incubation was interrupted at different time points by cooling on ice for 30 s. (**A**) Photograph of the 96-well plate after a 30-min incubation at 65°C, taken with a mobile phone. Wells with a yellow color indicate successful RT-LAMP amplification of a fragment of the SARS-CoV-2 *N* gene (using the N-A primer set). (**B**) Quantification of the red-to-yellow color change in all wells using spectrophotometric OD measurements. The color value at the given time points is quantified as the difference between the wavelengths of the two absorbance maxima of phenol red: ΔOD = OD_434 nm_ – OD_560 nm_. Yellow (positive) samples yield a ΔOD of about 0.3 to 0.4. Each line represents one sample. For each sample, the line color indicates the CT (cycle threshold) value obtained from RT-qPCR data (using the E-Sarbeco primers) (*15*). (**C**) Scatter plot of ΔOD values at the 30-min time point from (B) compared to CT values from RT-qPCR. Each dot is one sample (well).

To evaluate the colorimetric RT-LAMP assay, we needed to compare its sensitivity and specificity to a validated RT-qPCR method. We first used 95 RNA samples and performed RT-LAMP reactions using 1 μl of the isolated RNA in a reaction volume of 12.5 μl. We detected a red-to-yellow color change in 36 of the samples following an incubation of the reaction for 30 min at 65°C (Fig. 2A). To quantify the reaction, we used a plate scanner and measured the difference in absorbance (ΔOD) of the samples at 434 and 560 nm (corresponding to the absorbance maxima of the two forms of phenol red that were used in the assay as a pH-sensitive dye) at several time points. To visualize the data, we plotted the ΔOD values against incubation time and colored the time traces of individual samples according to the cycle threshold (CT) values obtained from the RT-qPCR test run in the clinical diagnostic laboratory (Fig. 2B). This RT-qPCR test was performed using a commercial diagnostic test kit containing a modified version of the E-Sarbeco primer set for the viral *E* gene suggested by Corman *et al. (**15*) and 10 μl of RNA isolated with an automated platform (QiaSymphony or QiaCube).

In a colorimetric RT-LAMP reaction, positive samples with a CT < 30 changed the color of the phenol-red dye within the first 30 min of the reaction. Samples with a CT > 30 either did not change their color or did so at time points > 35 min, simultaneously with a color change observed in some of the negative samples (Fig. 1). On the basis of this observation, we used the ΔOD value at 30 min to decide whether a sample was positive or negative. Plotting the ΔOD measurements versus CT values at the 30-min time point revealed that all patient samples with a CT < 30 showed a robust color change in the RT-LAMP test, whereas for samples with CT values between 30 and 35, a positive result was observed for only 1 of 10 samples (Fig. 2C). This suggested a detection limit of the colorimetric RT-LAMP assay corresponding to a CT ≈ 30 for RT-qPCR.

The RT-qPCR kit used was calibrated and a CT ≈ 30 corresponded to 1000 RNA molecules present in the reaction according to the certificate provided by the manufacturer (see Materials and Methods). The performance of each RT-qPCR run was validated using this as a positive control. Considering that 10 μl of isolated RNA was used for RT-qPCR, but only 1 μl for the RT-LAMP assay, a cutoff of CT ≈ 30 agreed well with the observed experimental sensitivity of approximately 100 RNA molecules for the RT-LAMP assay (Fig. 1A). Therefore, it appeared that the N-A primer set used for the RT-LAMP assay performed equally well with either IVT RNA or RNA samples isolated from the pharyngeal swab specimens.

In March 2020, at the beginning of the pandemic, the diagnostic laboratory that analyzed the pharyngeal swab samples by RT-qPCR validated all samples that tested positive with the *E* gene primer set in a second RT-qPCR using the *N* gene primer set, also of the Sarbeco sets of Corman *et al. (**15**).* When plotting RT-LAMP assay results against the CT values for the *N* gene primer set, we observed a sensitivity cutoff of around CT ≈ 35 (fig. S2A). Direct comparison of the CT values for the *E* gene and *N* gene primer sets for all samples revealed a difference of ~5.6 CT units (cycles) (fig. S2B). This suggested that the *N* gene primers were less sensitive than the *E* gene primers for detecting SARS-CoV-2 RNA by RT-qPCR. Similar differences have been observed previously for other primer sets, e.g., between the *E* gene primers and the RdRp-SARSr primers (*16**).*

For the RT-LAMP assay, we also tested the 1a-A primer set directed against ORF1a (*11*) and found this primer set to be less sensitive than the *N* gene LAMP primer set, with a sensitivity cutoff of CT ≈ 25 when plotted against *E* gene RT-qPCR–derived CT values (fig. S3). On the basis of these results, we decided to use the N-A primer set for the RT-LAMP assay and to compare our results with RT-qPCR performed with the E-Sarbeco primer set.

### Validation of the colorimetric RT-LAMP assay for SARS-CoV-2 RNA detection

**Fig. 3:**
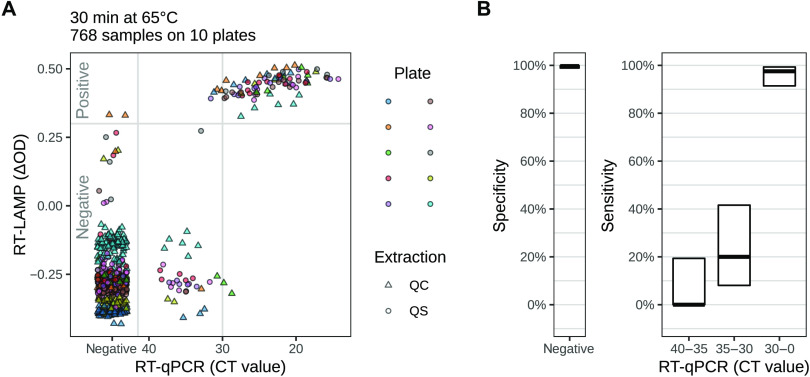
Detection of SARS-CoV-2 RNA using the RT-LAMP assay. (**A**) Scatter plot shows a comparison of RT-LAMP assay results and RT-qPCR results for RNA samples tested on 10 96-well plates. The RNA extraction method (QC, QiaCube, a column-based method; QS, QiaSymphony, a bead-based method) is indicated. The time point for measurement by the colorimetric RT-LAMP assay was 30 min after the start of the 65°C incubation. The 96-well plate shown in Fig. 2 is not included here. Table 1 shows numbers of samples stratified according to the results of the RT-LAMP and the RT-qPCR assays. (**B**) Sensitivity (right) and specificity (left) of the RT-LAMP assay [derived from data in (A) and Table 1] are shown. The specificity is the fraction of RT-qPCR–negative samples correctly identified as negative by the RT-LAMP assay. For sensitivity, the RT-qPCR–positive samples were stratified by CT values into three bins (as indicated by *x* axis labels), and for each bin, the sensitivity is given as the fraction of qPCR-positive samples in the respective CT bin that have also given a positive result in the RT-LAMP assay. The thick black lines indicate the values of these fractions (i.e., the specificity and sensitivity estimates); the black boxes indicate the corresponding 95% confidence intervals (Wilson’s binomial confidence interval). (See also table S2).

To determine the specificity and sensitivity of the RT-LAMP assay, we continued to analyze more RNA samples. We assayed a total of 768 RNA samples obtained on different days (fig. S1). Visualization of the RT-LAMP assay results 30 min after the start of the incubation at 65°C showed comparable behavior of the samples in a total of ten 96-well test plates (Fig. 3A and Table 1), indicating that the RT-LAMP assay was reproducible from day to day and from plate to plate.

**Table 1:** Shown is RT-qPCR and RT-LAMP testing of 768 clinical samples stratified into CT value bins (see Fig. 3A). Fig. 3B and table S2 show specificity and sensitivity values calculated from these numbers.

			**RT-LAMP**		
		**CT**	**Pos**	**Neg**	Sum
**RT-qPCR**	**Pos**	**0–25**	51	0	51
		**25–30**	28	2	30
		**30–35**	4	16	20
		**35–40**	0	16	16
	**Neg**	**Neg**	2	649	651
		Sum	85	683	768

The consistency of the results during the analysis confirmed a threshold of ΔOD > +0.3 as a robust measure to identify samples that were positive for SARS-CoV-2 RNA (Fig. 3A). RT-qPCR–positive samples with a CT < 30 scored positive in the RT-LAMP assay (79 of 81), whereas almost all samples with CT values between 30 and 40 scored negative (only 4 positive of 36) (Fig. 3B). This confirmed the sensitivity of the RT-LAMP assay for detection of SARS-CoV-2 RNA in samples corresponding to a CT < 30. We observed small differences between different plates on the exact sensitivity threshold, probably caused by slight variability in plate or reagent handling. We found two RT-qPCR–negative samples that scored positive in the RT-LAMP assay (Fig. 3A and Table 1) and one sample that scored just below the ΔOD cutoff of +0.3. The overall specificity of the RT-LAMP test was 99.7% (Wilson’s 95% confidence interval: 98.9 to 99.9%), and the sensitivity for samples with CT < 30 on RT-qPCR was 97.5% (Wilson’s 95% confidence interval: 91.4 to 99.3%) (Fig. 3B and table S2).

### Multiplexed sequencing of RT-LAMP reaction products

**Fig. 4:**
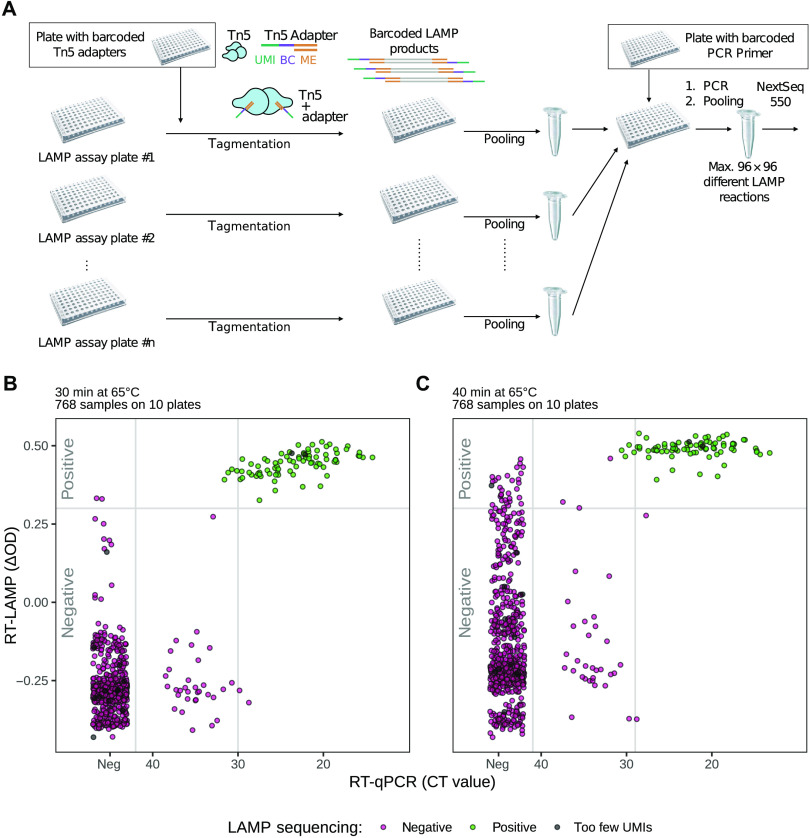
Multiplexed sequencing of RT-LAMP reaction products (LAMP-sequencing). (**A**) Workflow for LAMP-sequencing is shown. A plate of 96 barcoded (BC) adapters with unique molecular identifiers (UMIs) and mosaic ends (ME) was used as a seed plate for Tn5 tagmentation of all RT-LAMP reaction products. After tagmentation, each plate was pooled individually, followed by removal of excess adapters using size selection. Each pool of tagmentation products was then amplified using primers with plate-specific barcodes, and the PCR products were analyzed by Illumina sequencing. (**B**) Comparison of the outcome of the three assays: LAMP-sequencing (purple, negative; green, positive; gray, too few UMIs), RT-LAMP (after 30-min incubation, *y* axis), and RT-qPCR (*x* axis). Each dot represents one sample. If a substantial number of the sequencing reads contained SARS-CoV-2 RNA, the sample was called positive (green), if not, then it was called negative (purple). For some samples (gray), no LAMP-sequencing call could be made due to too few UMIs. (See also Table 2). (**C**) Although the RT-LAMP assay was scored after a 30-min incubation at 65°C (left), LAMP-sequencing was performed only after the samples had been incubated for another 10 min (15 min for one plate). This panel shows the RT-LAMP assay outcome (*y* axis) scored after the full incubation time, whereas the RT-qPCR CT values (*x* axis) and LAMP-sequencing results are the same as in (B).

Our results indicated that the colorimetric RT-LAMP assay enabled robust identification of positive samples after a 25- to 30-min incubation at 65°C. Validation of positive results, however, required confirmation that the RT-LAMP reaction led to the amplification of viral sequences. To analyze the sequences of many RT-LAMP reaction products, we established multiplexed sequencing of RT-LAMP products (LAMP-sequencing). LAMP-sequencing is based on Tn5 transposase tagmentation (*17*) and sample barcoding. Tagmentation enables fragmentation and direct adapter ligation of DNA samples for analysis by next-generation sequencing. We used a set of 96 barcoded adapters for tagmentation to barcode the RT-LAMP reaction products in each 96-well plate. After tagmentation, all barcoded fragments from each plate were pooled and size-selected by bead purification to remove excess adapters. A second set of barcoded primers, one per plate-pool, was then used to amplify the tagmented RT-LAMP fragments. Last, all amplified pools were combined for analysis using one next-generation sequencing run where the origin of each DNA fragment was specified by the two barcodes (Fig. 4A).

Of the LAMP-sequencing reads obtained, 98% mapped either to the part of the viral genome targeted by the RT-LAMP primers (80.6%) or contained short k-mers derived from primer sequences (17.4%) (fig. S4). This indicated that LAMP-sequencing amplified the targeted sequences. Reads containing only primer sequences were likely to be the result of spurious amplification products as these were also formed in the absence of input RNA (Fig. 1). For quantification of individual LAMP reactions, we classified reads according to whether or not they contained viral sequences, which were not directly covered by the primers (orange segments in fig. S4A), and counted the reads for each sample (as specified by its barcode combination) (fig. S4B). For 754 of the 768 samples, we obtained enough reads to make a call (fig. S5). For the 754 samples that underwent successful LAMP-sequencing, the results confirmed all samples that scored positive on the RT-LAMP assay with a CT < 30 (Fig. 4B and Table 2). For the two samples with a negative RT-qPCR result that scored positive on the RT-LAMP assay (Fig. 3), the LAMP-sequencing call agreed with the RT-qPCR result and thus corrected the RT-LAMP result.

**Table 2:** Summary of LAMP-sequencing results. The cross tabulation of RT-qPCR and RT-LAMP assay results shown in Table 1 have been split into samples where sequencing of RT-LAMP reaction products (LAMP-sequencing) was positive (Pos), negative (Neg), or inconclusive (too few reads) (see also Fig. 4).

					**RT-LAMP**		
				**CT**	**Pos**	**Neg**	Sum
**LAMP-** **sequencing**	**Pos**	**RT-qPCR**	**Pos**	**0–25**	49	0	49
				**25–30**	28	0	28
				**30–35**	4	0	4
				**35–40**	0	0	0
			**Neg**	**Neg**	0	0	0
	**Neg**	**RT-qPCR**	**Pos**	**0–25**	0	0	0
				**25–30**	0	2	2
				**30–35**	0	16	16
				**35–40**	0	16	16
			**Neg**	**Neg**	2	637	639
	**Too few reads**	**RT-qPCR**	**Pos**	**0–25**	2	0	2
				**25–30**	0	0	0
				**30–35**	0	0	0
				**35–40**	0	0	0
			**Neg**	**Neg**	0	12	12
				Sum	85	683	768

LAMP-sequencing was performed using the RT-LAMP samples after a prolonged incubation of 40 min at 65°C. At this time point, many of the negative samples and also samples with a CT between 30 and 40 had turned yellow. LAMP-sequencing eliminated all of these samples (Fig. 4C). This indicated that even for the RT-qPCR–positive samples with a CT between 30 and 35, the color change that took place at time points > 30 min was caused by spurious amplification products and not by late amplification of viral sequences. These results therefore confirmed that LAMP-sequencing was able to assess the results of multiple RT-LAMP reactions in parallel and to identify false-positive samples in the colorimetric RT-LAMP assay.

### A swab–to–RT-LAMP assay without RNA isolation

RNA isolation is time consuming, costly, and depends on reagents with potentially limited supply during a pandemic. Alternative, noncommercial solutions for RNA isolation, e.g., using silica gel matrix or magnetic beads, require specialized knowledge and cannot be implemented easily for point-of-care or decentralized screening.

Several reports have indicated that RT-qPCR (*18*–*20*) and RT-LAMP assays (*21*, *22*) are compatible with direct testing of nasopharyngeal and oropharyngeal swab specimens without a prior RNA purification or extraction step. To establish an RT-LAMP assay that could test unprocessed specimens (swab–to–RT-LAMP assay), we first assessed the stability of naked RNA in swab specimens that were collected in Amies medium. We titrated defined numbers of IVT RNA molecules of the SARS-CoV-2 *N* gene into swab samples from COVID-19–negative control subjects. We tested different conditions, particularly the influence of detergent (to inactivate the virus) and heat (to denature the capsid and release the viral RNA as well as inactivate the virus) (figs. S6 and S7, and data file S1). Consistent with previous reports about other RNA viruses (*23*–*25*) and tests using heat inactivation of swab specimens for direct RT-qPCR assays (*26*), these experiments established that native swab specimens and heat-treated swab specimens were compatible for detection of SARS-CoV-2 RNA in swab samples from infected individuals.

### Testing clinical samples with the swab–to–RT-LAMP assay

**Fig. 5:**
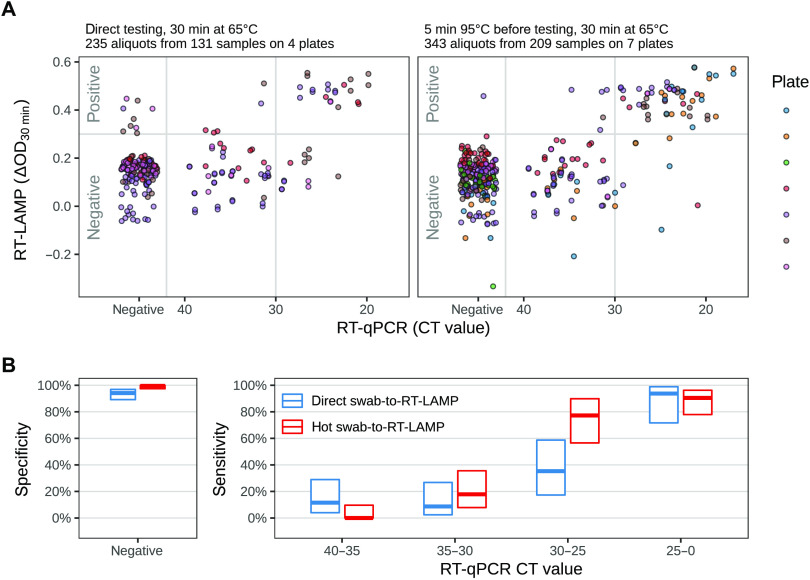
Swab–to–RT-LAMP assay of clinical pharyngeal swab samples. (**A**) Skipping a prior RNA isolation step, pharyngeal swab samples were subjected to the RT-LAMP assay either directly (left) or after 5 min of heat treatment at 95°C (right). For each sample, scatter plots are used to compare the swab–to–RT-LAMP assay results (ΔOD values) with the results of RT-qPCR (CT values). The measurement time point was 30 min after the start of the 65°C incubation. (**B**) Shown is the sensitivity (right) and specificity (left) of the swab–to–RT-LAMP assay [derived from the data in (A)] using the decision threshold indicated by the horizontal gray line in (A). Specificity and sensitivity values (thick lines) are shown with their 95% confidence intervals (boxes) as in Fig. 3, with blue indicating the direct swab–to–RT-LAMP assay and red indicating the hot swab–to–RT-LAMP assay. (Also see table S3).

On the basis of these preliminary experiments, we decided to use swab samples either directly without any treatment (direct swab–to–RT-LAMP assay) or after heat treatment for 5 min at 95°C (hot swab–to–RT-LAMP assay). As an additional precaution, we kept the samples in the cold (using an ice-cold metal block) whenever possible. For testing large numbers of clinical samples, we performed the RT-LAMP assay using several 96-well plates. In total, we tested 209 different samples using the hot swab–to–RT-LAMP assay, and of these, 131 samples also were tested by the direct swab–to–RT-LAMP assay. Many samples were tested twice but using aliquots withdrawn at different time points (usually within 24 hours) from the swab samples stored at 4°C. This resulted in 235 direct swab–to–RT-LAMP assay measurements and 343 hot swab–to–RT-LAMP assay measurements (Fig. 5A). The hot swab–to–RT-LAMP assay detected a color change in the majority of samples with a CT < 30 with high sensitivity, whereas the direct swab–to–RT-LAMP assay only exhibited a high sensitivity for samples with a CT < 25 (Fig. 5 and Table 3). The heat treatment rendered the RT-LAMP assay more stringent as it reduced false positives and more sensitive for samples with a CT of 25 to 30. We found that some positive samples did not induce a color change but did so when assayed a second time. We therefore would recommend running this assay using technical duplicates.

**Table 3:** Shown is RT-qPCR and RT-LAMP testing of 592 clinical samples stratified into CT value bins (see Fig. 5A). Fig. 5A and table S3 show specificity and sensitivity values calculated from these numbers.

**Hot swab–to–RT-LAMP**				**RT-LAMP**		
			**CT**	**Pos**	**Neg**	**Sum**
	**RT-qPCR**	**Pos**	**0–25**	38	4	42
			**25–30**	17	5	22
			**30–35**	5	23	28
			**35–40**	0	36	36
		**Neg**	**Neg**	1	214	215
			**Sum**	61	282	343
**Direct swab–to–RT-LAMP**				**RT-LAMP**		
			**CT**	**Pos**	**Neg**	**Sum**
	**RT-qPCR**	**Pos**	**0–25**	15	1	16
			**25–30**	6	11	17
			**30–35**	2	21	23
			**35–40**	3	23	26
		**Neg**	**Neg**	9	144	153
			**Sum**	35	200	235

### Heterogeneity of specimen pH in the swab–to–RT-LAMP assay

**Fig. 6:**
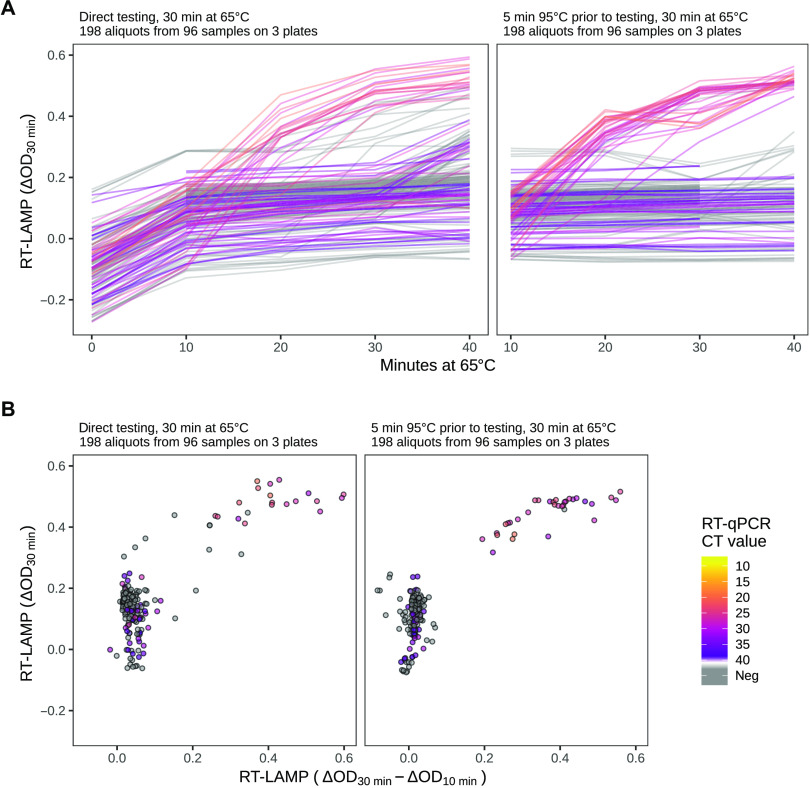
Colorimetric readouts of the swab–to–RT-LAMP assay over time. (**A**) The colorimetric readouts (ΔOD) for the direct (left) and hot (right) swab–to–RT-LAMP assays were assessed every 10 min. Heterogeneity is notable at the early time points. ΔOD values at the zero time point were not measured for the hot swab–to–RT-LAMP assay. Also, the 40-min time point was not available for one plate. The kink in some lines at 30 min (right) was due to a transient equipment malfunction. (**B**) Comparison of two scoring schemes. The readout used in Fig. 5 to score the direct (left) and hot (right) swab–to–RT-LAMP assays, namely, ΔOD at 30 min, is shown on the *y* axis, and compared to an alternative score, namely, the difference between the ΔOD signals at 30 min and at 10 min after the start of incubation, shown on the *x* axis. The latter shows better separation between positive and negative samples.

Comparison of the results of the direct swab–to–RT-LAMP assay with the RT-LAMP assay using isolated RNA revealed a much broader distribution of the ΔOD measurements in negative samples (Fig. 5A versus Fig. 3A). This was likely due to a sample-specific variability that influenced the starting pH in the LAMP reaction. This might have affected the interpretability of the measurement at 30 min (ΔOD_30min_). We investigated how this pH shift influenced the RT-LAMP assay. For three plates, the data acquired for the RT-LAMP assay also included measurements for the 10-min time point (ΔOD_10min_) (Fig. 6A). We plotted the change of the ΔOD between the 10- and 30-min time points (i.e., the difference ΔOD_30min_ – ΔOD_10min_, corresponding to the slope of the lines) versus ΔOD_30min_ (Fig. 6B). This removed the variability of the values for samples that did not change their color (negative samples) and permitted a better separation of the positive from the negative samples.

We noticed that the pH variability depended on the sample volume used for the RT-LAMP assay and the composition of the medium used for the swabs. For swabs in Amies medium (which was used for the clinical samples in this study), an RT-LAMP assay containing 1 μl of sample in a total volume of 20 μl was optimal. Our results obtained using native and heat-treated swab specimens suggested better performance when using heat treatment of swab specimens before running the RT-LAMP assay.

## DISCUSSION

Here, we evaluated the use and suitability of the RT-LAMP assay for the detection of SARS-CoV-2 infection. We also developed LAMP-sequencing as a fully scalable alternative to colorimetric or fluorometric analysis of DNA amplification reactions. Our results indicate that whereas the RT-LAMP assay using the N-A primer set is not sensitive enough to replace RT-qPCR in all applications, it does hold promise as a method for testing large numbers of samples.

We tested the RT-LAMP primer sets suggested by Zhang *et al. (**11*) and found that the N-A primer set for the *N* gene worked better than the 1a-A primer set for ORF1a. For samples with a CT ≤ 30 as measured by RT-qPCR with E-Sarbeco primers, we found overall satisfactory sensitivity and specificity values for SARS-CoV-2 RNA detection by the RT-LAMP assay using RNA samples isolated from pharyngeal swab specimens (Fig. 3 and Table 1). For samples with CT > 30, the RT-LAMP assay was much less sensitive. However, there is debate about which CT value for a positive RT-qPCR result should be considered clinically relevant. Vogels *et al. (**16*) indicate that a CT value above 36 corresponds to less than 10 molecules of RNA.

On the basis of our data, we conclude that the colorimetric RT-LAMP assay would be suitable for identifying individuals with a high or moderate SARS-CoV-2 viral load. On the other hand, for those with a low viral load (at the onset of illness or during later stages of the disease), the sensitivity of the RT-LAMP assay, in its current implementation using the N-A primer set, is insufficient to detect a SARS-CoV-2 infection. A number of other LAMP primer sets have been proposed and initially tested (*21*, *27*, *28*), showing that optimized primers and the use of combinations of primer sets hold promise to further increase the sensitivity of the RT-LAMP assay for detecting viral genomes. Furthermore, alternative sample types, e.g., sputum or stool (*29*), might be more reliable. One promising lead for future applications is the exploration of the hot swab–to–RT-LAMP assay using saliva specimens, although the relative sensitivity compared to using pharyngeal swab specimens is currently unclear (*30*–*33*). Compatibility of the RT-LAMP assay with direct saliva specimens has been shown using spike-in experiments (*22*, *34*).

Although faster and more convenient, the direct swab–to–RT-LAMP assay was less sensitive and less robust than the RT-LAMP assay using isolated RNA. To increase robustness, various treatments of crude swab samples have been described previously [reviewed in (*35*)], many of which require additional processing of the samples, for example, by pipetting or by adding proteinase K to degrade contaminating proteins. Rabe and Cepko (*22*) have suggested using cheap silica preparations and new sample inactivation protocols to enrich the RNA before the RT-LAMP assay, but this would complicate the simple swab–to–RT-LAMP assay workflow.

Last, our analysis found that a short heat treatment of 5 min at 95°C, which poses minimal additional handling steps, did not destroy the RNA but rather stabilized it and this improved the sensitivity and specificity of the swab–to–RT-LAMP assay (Fig. 5). The heat likely helped to homogenize the sample, to inactivate ribonucleases (RNAses), and to break up the viral capsid to release the viral RNA. Overall, our data demonstrate the feasibility of using a swab–to–RT-LAMP test and suggest applications especially in scenarios where RNA isolation is not available, e.g., in resource-poor settings. In such cases, the hot swab–to–RT-LAMP assay seems a good option given that the direct swab–to–RT-LAMP assay yields a number of false positives due to spurious amplification (*14*).

Although spike-in experiments with IVT RNA can be informative, we have experienced clear differences when comparing such experiments to those using clinical RNA samples isolated from swab specimens (figs. S6 and S7, and data file S1). We therefore recommend validating any new proposed rapid SARS-CoV-2 diagnostic test using “real-life” clinical samples including a large fraction of negative clinical samples. To overcome the problem of spurious amplification, an expanded oligonucleotide set that incorporates sequence-specific probes (*34*) or a CRISPR/Cas12a–based approach (*36*) could be used. However, these applications have yet to be tested with large numbers of diverse clinical samples.

There are several differences between the RT-LAMP assay and RT-qPCR. First, RT-qPCR requires a thermocycler to conduct the DNA amplification reaction, which is an expensive instrument, whereas isothermal incubation of RT-LAMP reactions can be conducted using a simple water bath or a heating block. This makes the RT-LAMP assay more amenable for point-of-care applications. Second, the reagents for the RT-LAMP assay are different from the ones used for RT-qPCR and are supplier independent. According to the supplier of the RT-LAMP reagents used in this study (New England Biolabs), production of RT-LAMP reagents can be easily ramped up to satisfy high demand. Third, the RT-LAMP assay, when combined with LAMP-sequencing, is suitable for analyzing large numbers of RT-LAMP reactions owing to the fully scalable DNA barcoding strategy. In contrast, there are several hurdles to scaling up RT-qPCR assays, the major hurdle being the need for a large number of thermocyclers. The RT-LAMP assay overcomes this problem and therefore will be a more scalable method for mass testing.

### Application of RT-LAMP and LAMP-sequencing for SARS-CoV-2 testing

With its good sensitivity for samples up to CT ≈ 30, the colorimetric RT-LAMP assay has several advantages: It is fast, inexpensive, and it can be evaluated without any equipment. RT-LAMP reactions also appear to be less sensitive to contaminants in the samples than RT-qPCR, but care has to be taken that the samples used do not alter the pH as the colorimetric RT-LAMP assay is performed under conditions of weak pH buffering. Some clinical samples contain contaminants that can lead to acidification of the reaction independent of the presence of a template RNA if too much sample is added. Diagnostic RT-qPCR tests usually include a technical internal control, i.e., another RNA species, which is spiked into all samples and which is detected independent of the gene of interest to safeguard against the possibility of a general reaction failure within a sample tube. It would be desirable to have a similar precaution for the RT-LAMP assay. A multiplexed fluorescence readout might provide this (*34*) but comes at the expense of the simplicity of a colorimetric readout.

Our particular implementation of deep sequencing to analyze many RT-LAMP reactions simultaneously uses two sets of barcoded primers and is fully scalable so that, in one sequencing run, many thousands of LAMP reactions can be quantitatively analyzed for the presence of viral genomic sequences. Although we used Illumina dye sequencing, more scalable sequencing technologies, such as Oxford Nanopore Technologies sequencing, could be used for amplicon sequencing and counting (*37*). The workflow shown here uses LAMP-sequencing as a validation and backup procedure to double check the results of the colorimetric RT-LAMP assay. However, LAMP-sequencing could also facilitate scale-up of the workflow for direct analysis of many thousands of samples in an efficient manner, provided that an infrastructure is established that allows the collection of such samples. Thus, LAMP-sequencing could become an important part of workflows for routine testing of large populations.

Schmid-Burgk *et al.* (*38*) proposed decentralized RT-LAMP assays using combinatorial primer barcoding and centralized mass analysis of RT-LAMP products by next-generation sequencing as a means to scale-up testing. Although this poses additional challenges in generating the individualized RT-LAMP assay reagents, it would simplify sample handling on the analytical side and it can be easily combined with the barcoding strategy shown here.

There are several limitations to our study. We used surplus RNA sample material from a diagnostic laboratory rather than newly collected clinical samples. The criteria for testing individuals may have influenced cohort characteristics and hence our findings. It is not clear yet how well viral load as indicated by CT values from RT-qPCR assays informs about the degree of infectivity of an individual with a SARS-CoV-2 infection. Therefore, we cannot say how our findings on the sensitivity of the RT-LAMP assay in comparison to RT-qPCR would translate into sensitivity for detecting infectious individuals who are shedding SARS-CoV-2 virus. Moreover, the measured viral load does not indicate the course of a SARS-CoV-2 infection, as even individuals with a very low measured viral load can still develop severe symptoms of COVID-19 disease. This may be, in part, because the viral load in a clinical sample taken from a specific site such as the pharynx is not representative of the overall viral burden that an infected individual carries.

We used LAMP-sequencing to validate the RT-LAMP assay results and did not use it as a diagnostic tool. LAMP-sequencing is dependent on the sensitivity of the RT-LAMP reaction as it cannot detect false negative results caused by a failure of the RT-LAMP assay to amplify viral RNA. Also, reagents such as the primer sets for the RT-LAMP assay may be subject to production-dependent quality fluctuations. Therefore, all reagents must be precisely validated (batch control) before using an RT-LAMP assay diagnostically.

Application of the RT-LAMP assay has great potential, even more so as more sensitive primer sets become available. The RT-LAMP assay and LAMP-sequencing could offer scalable testing that would be difficult to achieve with conventional RT-qPCR–based tests. For example, the RT-LAMP assay could be used for regular testing of a whole workforce or in sentinel testing, ideally combined with simplified sample collection, e.g., in the form of saliva samples. The RT-LAMP assay and LAMP-sequencing extend the range of available test methods and complement individual tests and pooled tests based on RT-qPCR (*39*) with a faster, simpler, and potentially more cost-effective test method.

## MATERIALS AND METHODS

### Study design

The intent of this study was to develop a clinical method for detecting SARS-CoV-2 RNA in RNA samples isolated from pharyngeal swab specimens from individuals being tested for COVID-19. We used pseudo-anonymized surplus RNA sample material that had been collected for clinical diagnosis of SARS-CoV-2 infection by RT-qPCR carried out by the diagnostic laboratory of Heidelberg University Hospital. Such reuse of material is in accordance with German regulations, which allow development and improvement of diagnostic assays using patient samples collected specifically to perform the testing in question. Pharyngeal swab specimens provided to us were either collected through the nose (nasopharyngeal) or the mouth (oropharyngeal), or sometimes one swab was used to collect both.

Our study was designed to investigate the sensitivity and specificity of a colorimetric RT-LAMP assay and to evaluate its suitability as an alternative to RT-qPCR testing for detecting SARS-CoV-2 viral RNA in RNA isolated from pharyngeal swab specimens. This study was conducted in Heidelberg, Germany in March and April of 2020. The study was designed to first evaluate different existing primer sets for RT-LAMP reactions and to use them for (i) detection of SARS-CoV-2 RNA in RNA isolated from pharyngeal swabs and (ii) detection of SARS-CoV-2 RNA directly from swab specimens without prior RNA isolation. All RNA samples used were pseudo-anonymized surplus material from the Heidelberg University Hospital diagnostic laboratory, and RT-qPCR results for these RNA samples were retrieved from the laboratory’s database only after the samples had been analyzed by the RT-LAMP assay. The study design was to conduct RT-LAMP testing until sufficient samples (at least several hundreds) had been analyzed to obtain a conclusive result. We also designed a deep sequencing-based method to validate the outcome of the RT-LAMP reactions using a Tn5 transposase–based fully scalable barcoding strategy (LAMP-sequencing).

### Clinical sample handling

Specimens were collected as nasopharyngeal and oropharyngeal flocked swabs in Amies medium (eSwab, Copan Italia). The sample collection happened as part of the routine operation of Heidelberg University Hospital and at public testing stations set up by the City of Heidelberg (fig. S1). Collected samples were transported in sterile containers, delivered to the diagnostic laboratory within a few hours, and then examined directly or stored at 4°C until further processing. Samples were processed in a biosafety level 2 cabinet until inactivation by heat or mixing with a lysis buffer.

### RNA isolation and RT-qPCR

The standard diagnostic pipeline of the hospital laboratory was as follows: RNA was isolated from nasopharyngeal and oropharyngeal swab specimens using QIAGEN kits (QIAGEN, Hilden, Germany); either automated on the QIASymphony (DSP Virus/Pathogen Mini Kits) or QIAcube (QIAamp Viral RNA Mini Kits) devices or manually (QIAamp Viral RNA Mini Kits). Please note that the QiaCube uses a sample volume of 140 μl and an elution volume of 100 μl, whereas the QiaSymphony uses a sample volume of 200 μl and an elution volume of 115 μl. RT-qPCR for the quantification of the SARS-CoV-2 viral genome was performed using kits and reagents from TIB MOLBIO Syntheselabor, Berlin, Germany. The kits were used according to the manufacturer’s instruction and contained the primer/probe sets developed based on the published Sarbeco primer set (*15*). Per 20-μl reaction, the master mix contained 5.4 μl of RNAse free water, 4.0 μl of LightCycler Multiplex RNA Virus Master (Roche, Basel, Switzerland), 0.5 μl of LightMix Modular SARS and Wuhan CoV *E* gene (cat. no. 53-0776-96; TIB MOLBIOL Syntheselabor GmbH, Berlin, Germany) or LightMix Modular SARS and Wuhan CoV *N* gene (cat. no. 53-0775-96; TIB MOLBIOL), 0.5 μl of LightMix Modular EAV RNA Extraction Control (cat. no. 66-0909-96; TIB MOLBIOL), and 0.1 μl of reverse transcriptase enzyme (LightCycler Multiplex RNA Virus Master, Roche, Basel, Switzerland). The master mix (10 μl) was distributed per reaction into 96-well plates, and 10 μl of purified RNA was added per well. The performance of the RT-qPCR was validated using a positive control for the *E* gene. A total of 10^3^ molecules of *E* gene RNA per RT-qPCR reaction correspond to a CT ≈ 30.

### RT-LAMP primer design and positive control

The RT-LAMP primer sets used in this study have been designed by Zhang *et al. (**11*) against ORF1a and *N* gene and were synthesized by Sigma-Aldrich (synthesis scale, 0.025 μmol; purification, desalt; solution, water). The sequences and the concentrations of each oligonucleotide in the 10× primer mix used for the RT-LAMP assay can be found in table S1.

An RNA-positive control for the *N* gene was amplified from a short fragment from 2019-nCoV_N_Positive control plasmid [Integrated DNA Technologies (IDT), 10006625] with oligonucleotides T7-GeneN-Fragment.for and GeneN-Fragment.rev including the T7 promoter and a subsequent IVT with the MEGAscript T7 Kit (Invitrogen) purified using the RNeasy MinElute Cleanup Kit (QIAGEN).

### Liquid handling using 96-well plates and precautions taken to prevent contamination

To prevent cross-contamination, we have taken several precautions. The 10× primer mix was prepared with nuclease-free water (AM9937, Ambion) and stored in aliquots at −20°C. To set up an RT-LAMP test, the RT-LAMP master mix was prepared freshly immediately before the test at a separate workspace with a dedicated pipette set and filter tips. The 96-well PCR plate containing the RT-LAMP mix was covered with an Society for Biomolecular Screening (SBS) plate lid. To avoid mix-ups during sample addition through well-by-well pipetting, the RNA or swab specimens were first collected into a 96-well seed plate. The RNA was then added to the plate with the LAMP reagents at a dedicated workspace with a manual 96-channel pipettor (Liquidator 20 μl, Mettler Toledo) using filter tips. The RT-LAMP and the RNA seed plate were instantly sealed with an optically clear adhesive seal (GK480-OS, Kisker Biotech) and an adhesive aluminum foil seal (SL-AM0550, Steinbrenner Laborsysteme, Germany), respectively. If the product of an RT-LAMP reaction had to be analyzed by gel electrophoresis, the plate was opened with extreme caution at a separated post-LAMP workspace and loaded onto an agarose gel with a dedicated pipette.

### RT-LAMP assay

Assays were assembled in total reaction volumes of either 12.5 μl (for LAMP assays using isolated RNA) or 20 μl (for swab–to–RT-LAMP assays). Master mixes were prepared at room temperature for each reaction immediately before use with either 6.25 or 10 μl, respectively, of the WarmStart Colorimetric RT-LAMP 2X Master Mix (M1800, New England Biolabs) and 1.25 or 2 μl, respectively, of the 10× primer mix, filled up to 11.5 or 19 μl with nuclease-free water (AM9937, Ambion). Values given are for one reaction: For a 96-well plate, 100 times larger volumes were used, and the LAMP mix was distributed to the wells of a 96-well plate (4ti-0960/C, Brooks Life Sciences or 0030128672, Eppendorf) before pipetting 1 μl of sample into each well of the plate; for details, see previous paragraph. Plates were prepared immediately before use to limit exposure of the LAMP reagents to atmospheric CO_2_ (to prevent acidification of the reaction) and kept on an ice-cold metal block. Plates were sealed using a transparent adhesive foil (GK480-OS, Kisker Biotech), and the reactions were incubated in a PCR cycler at 65°C for 15 to 60 min with the lid heated to 75°C. To perform measurements at the indicated time points, the reactions were taken out of the PCR cycler and placed into an ice cold metal block for 30 s. This intensifies the color before the measurement. Photographs were taken with cell phone cameras or the scanner function of an office copying machine.

### Quantification of the RT-LAMP reaction

Absorbance measurements were performed with a Spark Cyto or Infinite M200 (Tecan) at 434 and 560 nm with 25 flashes. These two peaks from phenol red are strongly changing during the acidification of the reaction (434 nm absorbance is increased, 560 nm absorbance is decreased). To obtain a good readout of the color change, absorbance at 560 nm was substracted from the one at 434 nm. This difference was denoted ΔOD.

### Swab–to–RT-LAMP assay

For direct and hot swab–to–RT-LAMP assays, patient swab specimens were transferred first onto a 96-well seed plate. For the direct assay, we then transferred 1 μl of the specimen directly to 19 μl of LAMP mix per well in a ready-made 96-well PCR plate (0030128672, Eppendorf). The plate was sealed using a transparent adhesive foil (GK480-OS, Kisker Biotech) and kept on an ice-cold metal block. For the hot assay, we sealed the seed plate with a pierceable lid (4ti-0566/96, Brooks Life Sciences) and heated it in a PCR cycler for 5 min at 95°C (with the lid heated to 105°C). The seed plate was cooled down to 4°C on an ice-cold metal block. Afterward, 1 μl of the heat-treated patient specimens was quickly added to a second ready-made plate with 19 μl of LAMP mix per well. This plate was also sealed with transparent adhesive foil (GK480-OS, Kisker Biotech). Both plates were then incubated at 65°C for the LAMP reaction to proceed. For both swab–to–RT-LAMP assays, the PCR plates were briefly spun down and then incubated in a PCR cycler at 65°C for 10 to 60 min (with the lid heated to 75°C). To perform measurements at the indicated time points, the reactions were taken out of the PCR cycler and placed into an ice-cold metal block for 30 s.

### LAMP-sequencing method

Sequencing libraries for detecting viral sequences in RT-LAMP products were prepared by a modified Anchor-Seq protocol (*37*, *40*) using Tn5 transposase tagmentation instead of sonication for genomic DNA fragmentation (*17*). The relevant primers are summarized in table S4.

In detail, transposon adapters containing well-defining barcodes and unique molecular identifiers (UMIs) were annealed by mixing 25 μM oligos (P5-UMI-xi5001…5096-ME.fw, Tn5hY-Rd2-Wat-SC3) in 5 μM tris-HCl (pH 8), incubating at 99°C for 5 min, and slowly cooling down to 20°C within 15 min in a thermocycler. Transposons were assembled by mixing Tn5(E54K, L372P) transposase (100 ng/μl) [purified according to (*41*)] with 1.25 μM annealed adapters in 50 mM Tris-HCl (pH 7.5) and incubating the reaction for 1 hour at 23°C. Tagmentation was carried out by mixing 1.2 μl of the RT-LAMP product (~200 ng DNA) with 1.5 μl of loaded transposase in freshly prepared tagmentation buffer [10 mM [tris(hydroxymethyl)methylamino]propanesulfonic acid) (TAPS)] (pH 8.5), 5 mM MgCl_2_, and 10% (v/v) dimethylformamide] using a Liquidator 96 Manual Pipetting System (Mettler Toledo). The reactions were incubated at 55°C for 10 min. Reactions were stopped by adding SDS to a final concentration of 0.033%. Tagmented DNA of each plate was pooled and size-selected using a two-step AMPureXP bead (Beckman Coulter) purification to target for fragments between 300 and 600 bp. First, 50 μl of pooled reaction was mixed with 50 μl of water and bound to 55 μl of beads to remove large fragments. To further remove small fragments, the supernatant of this reaction was added to 25 μl of fresh beads and further purified using two washes with 80% ethanol before the samples were finally eluted in 10 μl of 5 mM tris-HCl (pH 8). One PCR per plate with 1 μl of the eluate and RT-LAMP–specific and Tn5-adapter–specific primers (P7nxt-GeneN-A-LBrc and P7-xi7001..7016, P5.fw) was performed using NEBNext Q5 HotStart polymerase (New England Biolabs) with two cycles at 62°C for annealing and 90 s elongation, followed by two cycles at 65°C for annealing and 90 s elongation, and 13 cycles at 72°C annealing and 90 s elongation. All PCR reactions were combined and 19% of this pool was size-selected for 400 to 550 bp using a 2% agarose/tris-acetate-EDTA gel and column purification (Macherey-Nagel). The final sequencing library was quantified by qPCR (New England Biolabs) and sequenced with a paired-end sequencing run on a NextSeq 550 machine (Illumina) with 20% phiX spike-in and 136 cycles for the first read, 11 cycles to read the 11-nt-long plate index (i7) and 20 cycles to read the 11-nt-long well index (i5) and the 9-nt-long UMI.

For trimming of the reads (i.e., removal of P7 Illumina adapter sequences), cutadapt (version 2.8) (*42*) was used. For validation of the origin of the sequence of the LAMP product (fig. S4A), 10^7^ reads were randomly selected and used for the analysis. Reads were mapped to the SARS-CoV-2 reference genome (NC_045512.2) (*43*), using bwa-mem with default settings (version 0.7.17-r1188) (*44*). Virus genome coverage was determined with the samtools depth command (version 1.10) (*45*). Using bwa-mem, 80.6% of reads could be mapped to the virus genome (fig. S4, B and C). To analyze the remaining sequences, a k-mer analysis using a custom script was performed. Using 9-mers, this matched 93.5% of the nonmapped reads with a maximal Levenshtein distance of two to one of the LAMP primers or their reverse complement sequences (fig. S4D). This is explained by the fact that LAMP products can consist of complex sequence rearrangements.

For classification of samples by LAMP-sequencing, reads were assigned to wells and counted using custom scripts. A read was considered as a match to SARS-CoV-2 *N* gene if at least one of three short sequences (~13 nt, marked orange in fig. S4A) not covered by RT-LAMP primers was found in the read, otherwise it was counted as unmatched. Sequencing reads were grouped by UMI and by position of the matched sequence with the aim of removing PCR duplicates. A sample was considered if more than 200 total UMIs were observed and called positive if more than 10,000 virus-matching UMIs were observed.

There is a very wide gap in the number of virus-matching reads between positive and negative samples (fig. S5A): The count is either below 7000 UMIs or above 45,000 UMIs. This is why we placed the decision threshold for scoring a sample as LAMP-sequencing positive within this gap. The fact that also RT-qPCR–negative samples give rise to some UMI counts containing viral sequences is explained by template switching of unattached adapters that remain in the reaction after tagmentation, but no cause for concern due to the wide gap between negative and positive samples.

For a few samples, we saw so few reads (less than 200 UMIs) that we suspected that the multiplexing had failed and excluded them from the results. As most of these were in the same row of the same plate, we analyzed these samples after LAMP-sequencing by gel electrophoresis (fig. S5B) to check for DNA content after RT-LAMP. We found that the gel results agree with the RT-LAMP outcome, indicating that the failure likely was caused later, probably during multiplexing.

### Statistical analysis

Except where otherwise noted, all data were analyzed with R (*46*) using the tidyverse (*47*) and ggplot2 (*48*) system or with GraphPad Prism. Sensitivity and specificity values were obtained from count tables as follows: Specificity of the RT-LAMP assay was calculated as the fraction of RT-qPCR–negative samples that were also negative in the RT-LAMP assay. Sensitivity for a given CT interval was calculated as the fraction of all samples with an RT-qPCR CT value in that interval that was positive in the RT-LAMP assay. In both cases, 95% confidence intervals were calculated by interpreting the fractions of counts as binomial rates and then using Wilson’s method for binomial confidence intervals as implemented in the R package binom (*49*). The R code used to perform analyses and produce figures can be found on GitHub, together with all data tables: https://github.com/anders-biostat/LAMP-Paper-Figures.

## SUPPLEMENTARY MATERIALS

stm.sciencemag.org/cgi/content/full/scitranslmed.abc7075/DC1 Materials and Methods Fig. S1. Design of the study. Fig. S2. Comparison of the RT-LAMP assay with CT values from RT-qPCR using primer sets E-Sarbeco and N-Sarbeco. Fig. S3. Comparison of the RT-LAMP assay using primer set 1a-A with CT values from RT-qPCR using primer set E-Sarbeco. Fig. S4. Analysis of LAMP-sequencing reads. Fig. S5. Sample classification with LAMP-sequencing. Fig. S6. RNA stability and detection limit of the RT-LAMP assay in pharyngeal swab specimens with IVT RNA. Fig. S7. Swab–to–RT-LAMP assay titration of positive COVID-19 specimens. Table S1. Sequences of primers and amplicons used in this study. Table S2. Sensitivity and specificity of the RT-LAMP test from Fig. 3B. Table S3. Sensitivity and specificity of the RT-LAMP test from Fig. 5B. Table S4. Primers used for LAMP-sequencing. Table S5. Overview of time requirements for various sample handling steps. Data File S1. Raw data for figs. S6 and S7.

## Appendix

View/request a protocol for this paper from *Bio-protocol*.
